# MonaGO: a novel gene ontology enrichment analysis visualisation system

**DOI:** 10.1186/s12859-022-04594-1

**Published:** 2022-02-14

**Authors:** Ziyin Xin, Yujun Cai, Louis T. Dang, Hannah M. S. Burke, Jerico Revote, Natalie Charitakis, Denis Bienroth, Hieu T. Nim, Yuan-Fang Li, Mirana Ramialison

**Affiliations:** 1 Faculty of IT, Monash University, Clayton, VIC Australia; 2 Australian Regenerative Medicine Institute, Monash University, Clayton, VIC Australia; 3 Southeast University, Nanjing, China; 4 Systems Biology Institute Australia, Clayton, VIC Australia; 5 Monash eResearch Centre, Monash University, Melbourne, VIC Australia; 6 Murdoch Children’s Research Institute, Parkville, VIC Australia

**Keywords:** Gene ontology, GO enrichment, Web services, Interactive visualisation, Semantic web

## Abstract

**Background:**

Gene ontology (GO) enrichment analysis is frequently undertaken during exploration of various -omics data sets. Despite the wide array of tools available to biologists to perform this analysis, meaningful visualisation of the overrepresented GO in a manner which is easy to interpret is still lacking.

**Results:**

Monash Gene Ontology (MonaGO) is a novel web-based visualisation system that provides an intuitive, interactive and responsive interface for performing GO enrichment analysis and visualising the results. MonaGO supports gene lists as well as GO terms as inputs. Visualisation results can be exported as high-resolution images or restored in new sessions, allowing reproducibility of the analysis. An extensive comparison between MonaGO and 11 state-of-the-art GO enrichment visualisation tools based on 9 features revealed that MonaGO is a unique platform that simultaneously allows interactive visualisation within one single output page, directly accessible through a web browser with customisable display options.

**Conclusion:**

MonaGO combines dynamic clustering and interactive visualisation as well as customisation options to assist biologists in obtaining meaningful representation of overrepresented GO terms, producing simplified outputs in an unbiased manner. MonaGO will facilitate the interpretation of GO analysis and will assist the biologists into the representation of the results.

**Supplementary Information:**

The online version contains supplementary material available at 10.1186/s12859-022-04594-1.

## Background

Gene Ontology (GO) [1] is widely used in biomedical sciences to mine large-scale datasets. GO enrichment is one of the most popular post-omics analyses for datasets generated by genomics, transcriptomics, proteomics and metabolomics assays. A myriad of web-based tools or software packages are available to perform GO enrichments or classification, including the popular tools Database for Annotation, Visualization and Integrated Discovery (DAVID) [2], Protein ANalysis Through Evolutionary Relationship (PANTHER) [3] and the long-established Gene Set Enrichment Analysis (GSEA) [4].

Inappropriate use of GO enrichment analyses can result in misleading targets and waste of resources, presenting massive hurdles to biologists [5]. For example, if several GO categories are predicted to be enriched above the statistical threshold, which of them should be displayed? Often arbitrary decisions are made such as keeping only the “top 5 most-enriched” as a figure in publications. In addition, the redundancy of GO terms due to its hierarchical nature makes visualisation of enrichment results difficult, and often “representative terms” (e.g. “inflammation” or “differentiation”) are arbitrarily chosen to represent broadly-related GO categories. The emerging field of visual analytics [6] and its increasing use in biomedicine [7] can bridge these challenges by harnessing human expertise to navigate the dense information typically presented in GO enrichment analyses, resulting in a meaningful representation of overrepresented GO terms.

We have developed MonaGO, a novel interactive online visualisation system for GO enrichment analysis results. MonaGO provides a coordinated interface that retains all information, yet remains intuitive, fluid, and easy to use for lay users. Therefore, MonaGO assists biologists in making informed decisions on which enriched terms should be displayed to allow a meaningful representation and interpretation of their datasets, without compromising on objectivity by arbitrarily choosing “representative terms”.

Several tools exist that provide visualisation for GO enrichment analysis results [8–10] but in addition MonaGO offers (1) on-the-fly exploration of GO terms clustering via chord diagram visualisation, (2) the ability to manually or systematically cluster GO terms interactively, in an intuitive and interactive interface.

### Implementation

MonaGO utilises a client–server architecture and it comprises two main parts: (1) a frontend client receiving inputs from users and visualising the data, and (2) a backend server responsible for processing data, querying database and producing data for visualisation. The client is mainly built in JavaScript and the server is built in Python.

The server consists of two Python modules. The first, server.py, utilizes *Flask 1*, a stable and scalable web application development framework. Specifically, when given a list of genes, this module sends a request to DAVID to obtain GO enrichment results. It also maintains a copy of the Gene Ontology hierarchy for visualising already enriched genes. Responses from DAVID are filtered and passed to the data-processing module. In addition, visualisation from a previously saved session can be restored by uploading a previously exported file, which already contains processed data. The *server.py* module parses the file and sends it to client for visualisation directly. Redundant server nodes were implemented using the round-robin load balancing scheme to improve multi-user responsiveness.

**Fig. 1:**
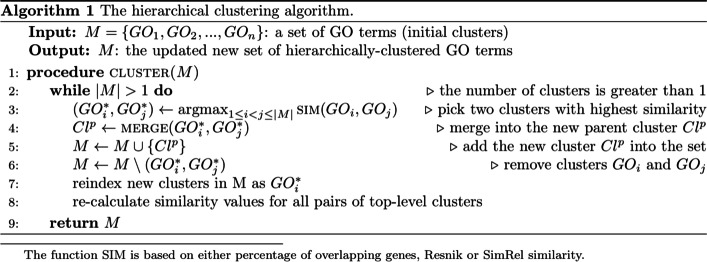
MonaGO’s hierarchical clustering algorithm (Algorithm 1) to produce the dynamic chord diagram

The second module, *dataprocess.py*, performs data processing tasks, including calculating cluster similarity, creating hierarchical clusters, and reordering clusters for visualisation. Specifically, a hierarchical clustering algorithm is employed to cluster GO terms into clusters according to one of three similarity metrics: the percentage of common genes between pairs of them (Jaccard similarity), the Resnik similarity [11] between GO terms, and the SimRel similarity [12] between GO terms. Algorithm 1 (Fig. 1) provides a more detailed description of the clustering process. In doing this, the algorithm computes the similarity score between two clusters (as represented by the function SIM) based on the user’s input. If the user chooses ‘percentage of common genes’ as the similarity measurement, the genes found in any of the GO terms in each cluster are compared, and the intersection is returned as a percentage. If the user chooses a semantic similarity (Resnik or SimRel), the similarity value is found for each combination of GO terms in the two clusters. The aggregate of these values is then returned based on the user’s choice for aggregate function (average, minimum or maximum).

Semantic similarities are calculated using the formulas described by Schlicker and Albrecht [13]. In order to evaluate these formulas, two databases are used; firstly, to count the number of annotations of each GO term we use the Universal Protein Knowledgebase (UniProtKB) [14] (updated 14/02/19). Secondly, to access the Gene Ontology hierarchy we use *go-basic.obo* (accessed 05/03/19).

The client functions as a receiver and visualisation platform. MonaGO.js serves as the main controller of functionalities. Dynamic and interactive graphics are generated using *D3.js*, a JavaScript library allowing great control over final visualisation results. Through the visual interface generated by the client, users can intuitively interact with the visualisation and download high-resolution images from MonaGO, in Portable Document Format (PDF), Portable Network Graphics (PNG) or Scalable Vector Graphics (SVG).

## Results and discussion

### MonaGO’s interface allows a user-friendly interactive display of GO enrichment results

**Fig. 2:**
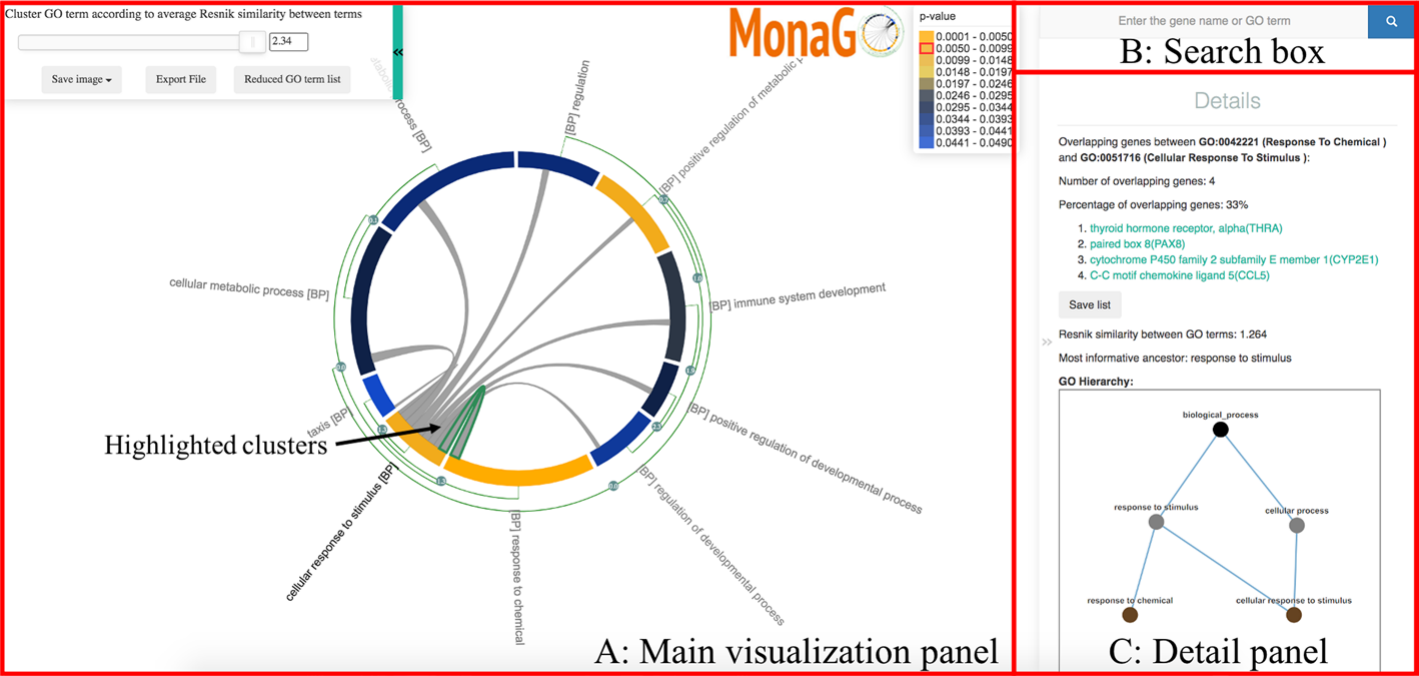
The main visualisation interface of MonaGO consisting of three components: **A** (left) the main visualisation panel on the left that shows the chord diagram of GO terms that can be hierarchically and dynamically clustered, **B** (top right) search box, and **C** (bottom right) the details panel with dynamic GO hierarchy visualisation

MonaGO supports three different ways of data entry: (1) submitting a list of genes to DAVID [2], one of the most widely used programs, to perform enrichment analysis in the background, (2) submitting gene lists and associated, pre-selected enriched GO terms for visualisation directly, and (3) importing a previously exported visualisation to restore it. MonaGO’s output options (Fig. 2) include high-resolution PNG or SVG images of a chord diagram (in the main visualisation) and the ontology hierarchy of a GO term (in the details panel), as well as JavaScript Object Notation (JSON) files that store the current state of the main chord diagram which can later be imported and restored in MonaGO.

The main visualisation interface (Fig. 2) comprises three main components: the main visualisation panel displays hierarchical clustering on a chord diagram, with each node representing a cluster of enriched GO terms (Fig. 2A), the “search box” panel allows browsing for specific terms or genes annotated by these terms (Fig. 2B), and a “details” panel displays further information on a specific GO term upon selection (Fig. 2C).

To provide biologists with a comprehensive representation of GO enrichment results, a chord diagram (centre of Fig. 2) is employed as an intuitive and compact way to visualise clusters of GO terms and similarity between them. Enriched GO terms are colour-coded based on their p-values and the lengths of their arcs are proportional to the numbers of genes-of-interest contained in them.

The green arcs parallel to the main chord diagram on the outside denote possible (hierarchical) clusters, and the number on an arc-node represents the percentage of common genes-of-interest between two nodes/clusters. The grey links inside the chord diagram connect pairs of GO term/clusters, and the presence of such a link denotes the existence of common genes-of-interest between them.

MonaGO helps reduce redundancy by hierarchically clustering similar GO terms in the main chord diagram. In MonaGO, enriched terms are ordered hierarchically, in order to allow collapse and expansion operations on the GO hierarchical clusters. Users can choose between three distance metrics for the initial clustering GO terms: percentage of overlapping genes or their semantic similarity (Resnik similarity [11] and SimRel [12]). When using Resnik and SimRel, users can choose between *average*, *minimum* or *maximum* options, based on the semantic similarity between each combination of individual terms in the GO clusters. *Average* takes the mean of all these similarities, and hence represents the distance between two areas of the GO hierarchy. Alternatively, *minimum* represents the distance between the farthest two nodes of the clusters and *maximum* the closest two nodes. Hence minimum considers the Most Informative Ancestor common to all GO terms in the clusters, whereas maximum considers the Most Informative Ancestor of any two terms.

Through this chord diagram display, users can easily cluster GO terms with overlapping descriptions as they wish, thereby reducing the information content. There are two ways to perform clustering: systematically and manually. Systematic clustering refers to automatically clustering GO terms based on a threshold of the similarity score within clusters. A slider at top left of the main component allows users to control this threshold, and GO terms are subsequently clustered such that the similarity score within the cluster is greater than the given threshold. In addition to this, users can adjust any of the clusters manually. Manual clustering refers to dynamically collapsing or expanding clusters, according to the hierarchy of enriched GO terms, within in the chord diagram by clicking the green arc-nodes. This action equates to setting at which threshold the parent node is selected to visualising the term, according to the hierarchical structure of the ontology. The option to cluster manually in conjunction with systematically allows the user to easily reduce the GO terms instantly based on a similarity score, and subsequently make further refined collapsing or expansions of the GO clusters, based on their own interpretation of the importance of each GO term.

**Fig. 3:**
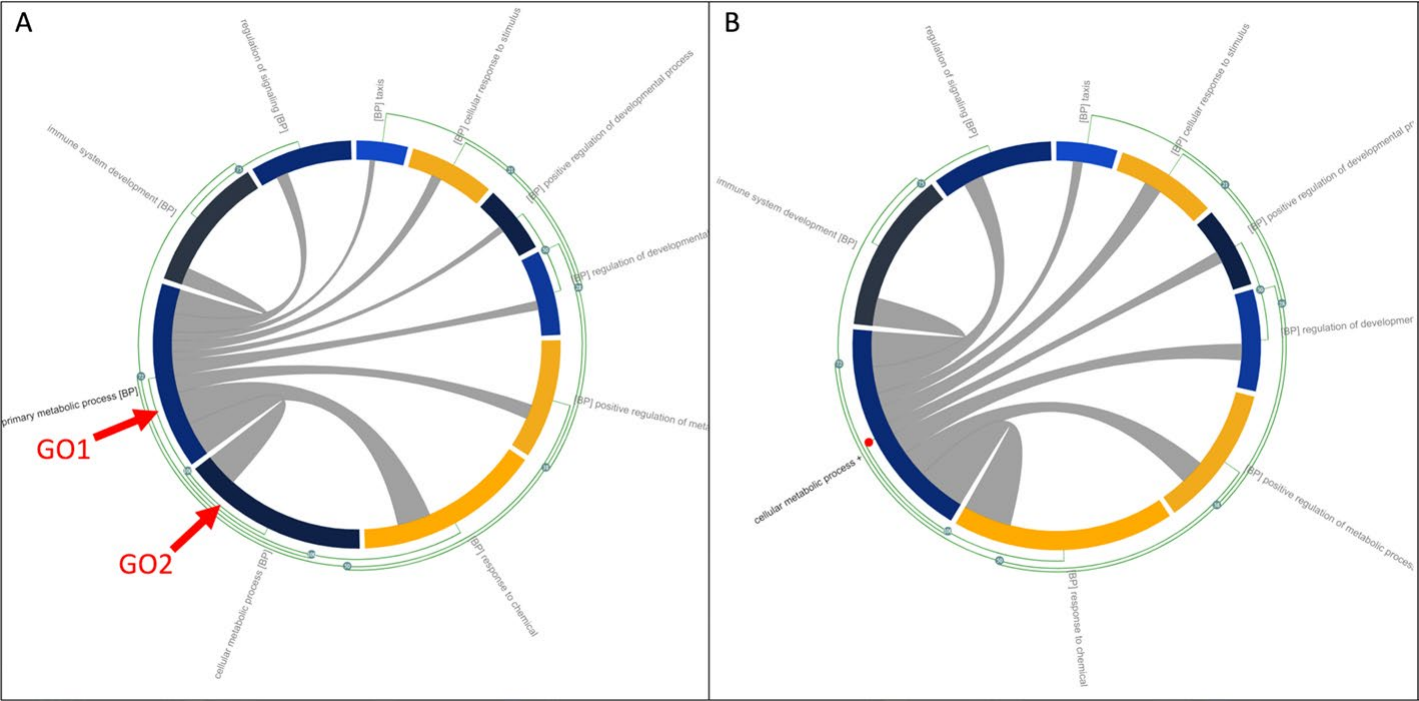
An example usage of the manual clustering feature of MonaGO which allows to dynamically collapse or expand nodes in the hierarchy of enriched terms: **A** the GO chord diagram before clustering where GO1 and GO2 are to be merged and, **B** the GO chord diagram after clustering

An example of manually collapsing and expanding clusters is shown in Fig. 3. As highlighted in Fig. 3A, GO1 and GO2 share 11 common genes, which amounts to 100% of their total genes. If a user considers GO1 and GO2 to be highly similar and wishes to group them, the number on the green arc between them can be clicked and thus create a cluster (Fig. 3B). Similarly, if the user considers both GO1 and GO2 necessary terms but they have been clustered systematically, they can click on the red dot that appears outside the cluster node shown in Fig. 3B. This will expand the cluster, reverting it back to Fig. 3A.

High-resolution images of the chord diagram and the GO hierarchy of a selected GO term in the details panel can be saved in three formats, PDF, SVG and PNG, by clicking the drop-down menu button “Save image” at the top left of the main visualisation panel.

Moreover, a JSON file storing the current state of the main chord diagram and data of GO enrichment results can be downloaded and later imported into MonaGO to restore the state of the visualisation for subsequent analysis.

The details panel shows, for a node/cluster on the chord diagram or a link inside the chord diagram, additional information about it that is complementary to the main chord diagram. For any given grey link (highlighted with a green outline, Fig. 2A), the panel displays (1) the number and percentage of shared genes between the two GO terms, (2) the list of these shared genes and (3) information of the semantic similarity between these terms (if chosen as similarity measure) including a diagram of the GO hierarchy (Fig. 2C). The hierarchy diagram can be expanded to full screen if needed, in order to view the graph more clearly [15].

Finally, the search box provides a convenient alternative way of finding genes and their associated GO terms by free-text search (Fig. 2B). GO terms annotating a matched gene are listed in the details panel as well as dynamically highlighted in the chord diagram for easy identification (Fig. 2A).

### MonaGO offers unique visualisation properties compared to existing tools

To assess MonaGO’s visualisation properties, we compared it to twelve well-known and highly-cited GO analysis systems which offer a visualisation platform (Table 1) for GO enrichment analysis. Systems such as DAVID [2] and PANTHER [3], provide a large number of analytical services where tables or simple graphs are used to display enrichment results. Other systems such as REduce and VIsualise Gene Ontology (REVIGO) [10], g:Profiler [16], Gorilla [17], WebGestalt [18], and Gene Ontology plot (GOplot) [19] are primarily visualisation systems dedicated to representing GO enrichment analysis results. MonaGO offers an ideal combination by providing a visualisation interface either based on existing results from GO enrichment analysis or performing GO enrichment from scratch through DAVID. Hence, where most of the systems accept GO terms (GOplot [19], REVIGO [10]) or genes (WebGestalt [18]), MonaGO offers three types of input, allowing a user to (1) submit gene lists and perform the enrichment using DAVID [8], (2) submitting GO terms with annotations directly, and (3) restoring previous visualisation results.

**Table 1:** Comparisons of MonaGO with existing GO analysis systems with visualisation capabilities

System	Interactive	Visualisation of enriched GO terms	Platform/dependency	Download/save data	Find similar terms/select important terms	Show relationship between GO terms	Flexible switching between pages	Flexible threshold (p-value, etc.)	Input list	Examples
MonaGO	Yes	Chord diagram	Browser	Yes	Yes	Yes	Yes	Yes	Genes, GO terms, previous visualisation	Yes
agriGO [24]	No	Node-link diagram	Browser	Yes	Yes	Yes	No (visualisation a subsequent step/page)	Yes	Genes, Probes	Yes
AmiGO [25]	No	Node-link diagram	Browser	Yes	Yes	Yes	No	Yes	Genes	No
BiNGO [20]	Yes	Node-link diagram	Cytoscape	Yes	Yes	Yes	Yes	Yes	Graph or gene list input	Yes
DAVID [2]	No	Clustering, map	Browser, programmatically (Java, Perl, Python)	Yes	Yes	Yes	No (several pages	No	Genes	Yes
GOEAST [21]	No	Node-link diagram	Browser	Yes	Yes	Yes	Yes	Yes	Probe set ID	No
GOplot [19]	No	Circle plot, chord plot	R	Yes	Yes	Yes	No	Yes	GO terms only	Yes
Gorilla [17]	No	Node-link diagram with highlights	Browser	Yes	No (abstract and redundant hierarchy)	Yes	No	Yes	Genes, proteins	Yes
g:Profiler [16]	No	Bar chart, Word cloud	Browser	Yes	Yes (by ranking)	No (matrix denotes common genes between GO terms)	No	Yes	Genes, proteins, probes	Yes
Metascape [26]	No	Node-link diagram, Bar chart	Browser, Cytoscape	Yes	Yes	Yes	Yes	No	RetSeq, Ensembl, Uniprot, UCSC	Yes
PANTHER [3]	Yes	Pie chart	Browser	Yes	Yes	Yes	No (several pages)	No	Genes, proteins	Yes (only format, no complete examples)
REVIGO [10]	Yes	Node link diagram, Treemap Word cloud, Scatterplot	Browser	Yes	Yes	Yes	Yes	Yes	GO terms	Yes
WebGestalt [18]	Yes	Node link diagram	Cytoscape	Yes	Yes	Yes (but no interactive details about common gene lists	Yes	Yes	Genes	Yes

Node-link diagrams are widely used (e.g. Biological Networks Gene Ontology (BiNGO) [20], Gene Ontology Enrichment Aanalysis Software Toolkit (GOEAST) [21], Gorilla [17], and WebGestalt [18]) when it comes to showing relationships between GO terms. However, the GO hierarchy or term-term relationships are not easily shown in such an approach. To address this limitation, MonaGO displays term-term similarity in a chord diagram while providing hierarchy and other information in the details panel. This split representation allows different levels of information to be displayed, while avoiding to clutter the interface. Some tools feature interactive visualisation outputs (DAVID [2], REVIGO [10], and WebGestalt [18]) by reloading the display after re-setting the parameters of interest (such as setting threshold). Other tools (g:Profiler [16], Gorilla [17], GOplot [19]) only provide static interfaces/images. MonaGO provides a truly interactive interface as the changes in the visualisation parameters are simultaneously reflected on the output display as the user modifies them.

### MonaGO’s interactive interface allows prioritisation of which enriched GO terms to display

MonaGO is one of a few GO visualisation tools that display the relationship between terms based on the number of common genes. To illustrate the advantages of MonaGO, we re-analysed our published datasets where we measured gene expression changes for three cells types (fibroblasts, neutrophils and keratinocytes) while reprogramming the cells into a pluripotent state [22]. In brief, genes sharing similar expression levels over five stages of reprogramming were clustered using c-means fuzzy clustering. GO term enrichment for selected clusters was performed using DAVID [8].

**Fig. 4:**
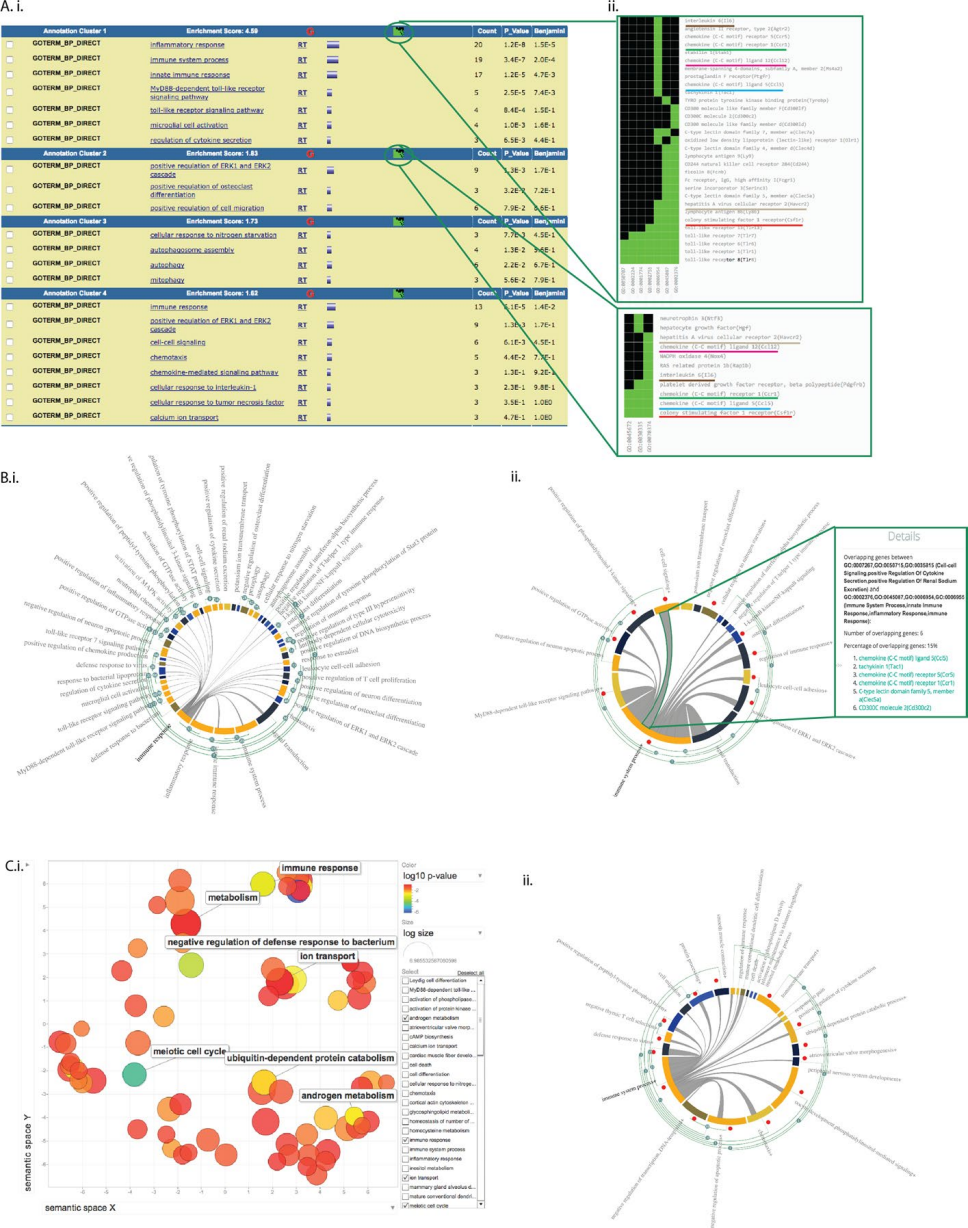
Using MonaGO to study functions of genes involved reprogramming fibroblasts to a pluripotent state. **A** List of clustered terms from GO enrichment of these genes using DAVID: **A.i** term clustering table; **A.ii** common genes display. **B** MonaGO clustering result of the same gene sets used in **A**, showing **B.i** clustering of the full set of terms; and **B.ii** manual clustering by node collapsing from fibroblast gene cluster 4 in Nefzget et al. 2017. **C** Visualisation of genes from 6 representative fibroblasts clusters by **C.i** REVIGO and **C.ii** MonaGO

DAVID’s default display output is a list of terms or cluster of terms (Fig. 4Ai). In this test dataset, several over-represented GO terms were found enriched. Due to the length of the list, it is thus not uncommon that only the most statistically significant terms or terms relevant to the biological question are retained, creating selection bias of GO terms (Fig. 4Ai). In contrast, MonaGO displays all over-represented terms (Fig. 4Bi) in a single view, which can be further systematically reduced into more generic clusters (Fig. 4Bii), using the overlapping number of genes as a threshold, knowledge of genes common to these clusters can be further capitalised to unravel the molecular mechanisms driving these enriched biological processes. In DAVID, this information is accessible through the cluster display mode (Fig. 4Aii), where genes shared between enriched GO terms within a cluster are listed as a static heatmap. In MonaGO, at any stage during the clustering process, the genes shared between the clusters are accessible in the chord diagram (Fig. 4Bii) which will assist in the interpretation of the data. For instance, in the test dataset, the most significant term “immune response” has been clustered under “immune system process”, however genes in this category are also involved in other biological processes. For example, out of 35 genes in the top category, six genes (*Ccl5*, *Tac1*, C*ccr5*, *Ccr1*, *Clec5a* and *Cd300c2*) are also involved in ‘cell–cell signaling’. MonaGO thereby allows the users to establish functional links between terms that are otherwise just presented as disjoint items in a list. Using the fibroblast dataset on REVIGO [10], reduction of number of GO terms is effective and visualisation of these similar GO terms is clear (Fig. 4Ci), based on hierarchy level and p-value. Similar clusters are retrieved through MonaGO (Fig. 4Cii), however the inclusion of common genes to GO clustering provides a unique perspective on the functional relationships between GO enriched terms.

In conclusion, MonaGO’s chord-diagram based interface allows an unbiased exploration of GO clustering results. By supporting systematic clustering of GO terms and displaying the relationships between the terms that are directly informed from the dataset, MonaGO produces meaningful representation of overrepresented GO terms in an unbiased manner.

### Clustering of GO terms by overlapping genes or semantic similarity simplifies the GO output and reveals novel functional properties

MonaGO offers two distance similarity measurement options to cluster the enriched GO terms in the chord diagram. We assessed the biological outcomes resulting from using Resnik semantic similarity versus percentage of overlapping genes, using an in-house curated list of zebrafish embryonic cardiac genes (Additional file 1). We used MonaGO to assess which biological functions compose the developmental circuitry of the heart.

**Fig. 5:**
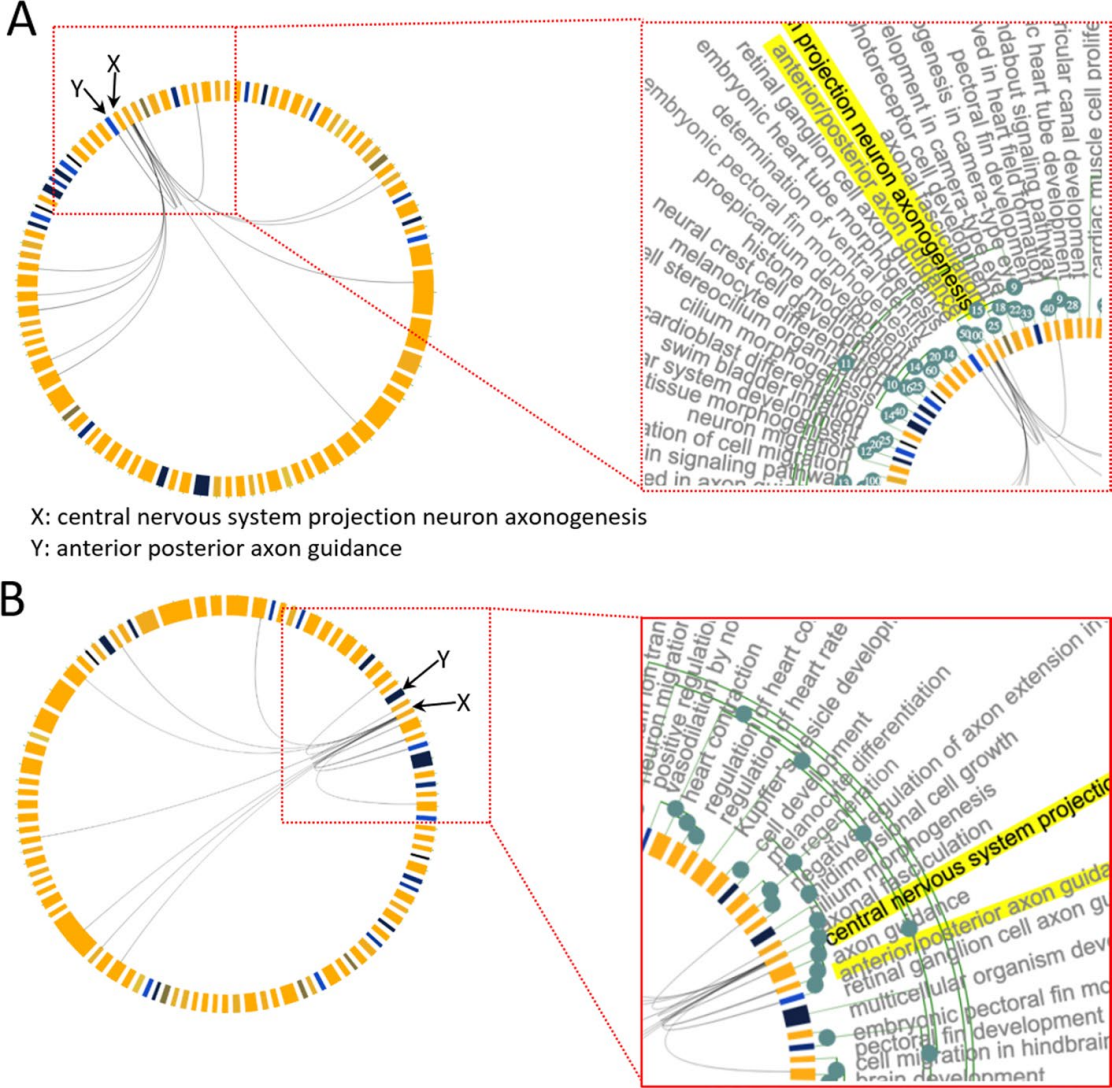
**A** Section of MonaGO’s visualisation with set of cardiac genes from zebrafish and overlapping genes as distance measurement. The GO terms ‘central nervous projection neuron axonogenesis’ and ‘anterior/posterior axon guidance’ showing 100% of overlapping genes, are highlighted in yellow. **B** Same visualisation as **A** but using Resnik similarity as distance measurement instead

**Fig. 6:**
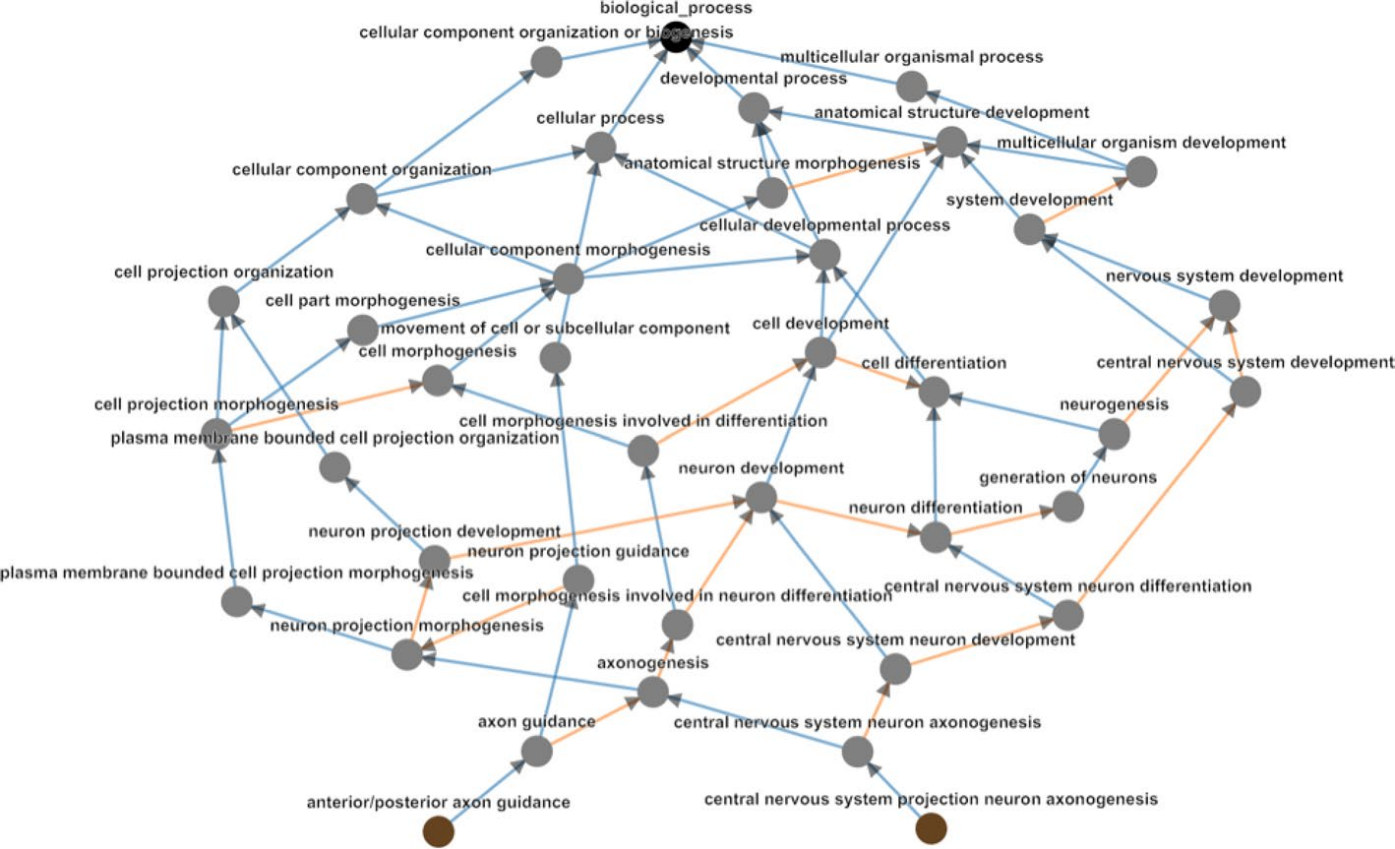
GO hierarchy between the Biological Process terms ‘central nervous system project neuron axonegenesis and ‘anterior/posterior axon guidance.’ These have Resnik similarity of 3.638, with their Most Informative Ancestor being ‘axonogenesis.’

Running MonaGO using “official gene symbol” as the identifier and ‘percentage of overlapping genes’ as the distance measurement allows to build a workable shortlist of biological functions that are over-represented in this gene set. As an example, running the cardiac gene set in this mode identified two different neighbouring terms ‘central nervous system projection neuron axonogenesis’ and ‘anterior/posterior axon guidance’ sharing 100% of overlapping genes (Fig. 5A), hence suggesting that despite being described by different names, these two categories may represent the same function. This is further confirmed by reperforming this test using ‘Resnik similarity (average)’, where these two terms are still grouped into the same cluster (Fig. 5B). Investigation of the GO hierarchy shared between the terms, which is also a feature of MonaGO, explains that their functional similarity pertains to ‘axonogenesis’ (Fig. 6). Hence biologists can be confident that grouping the terms into a single node is a valid operation which also helps reduce information repetition.

**Fig. 7:**
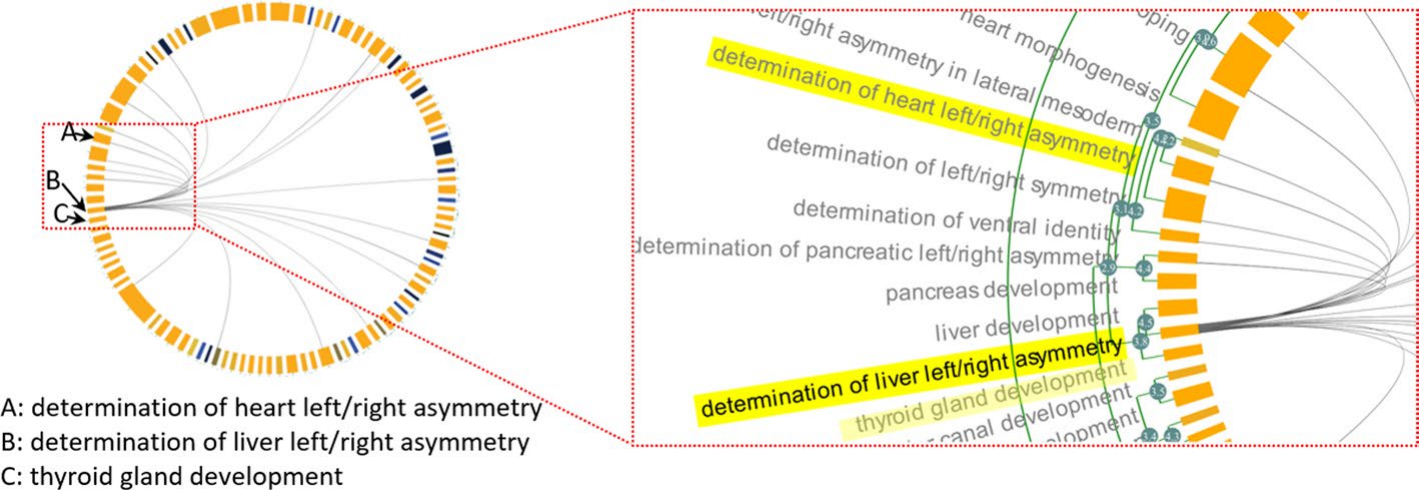
Section of MonaGO visualisation with set of cardiac genes from zebrafish and Resnik similarity as distance measurement. The GO terms ‘liver development’, ‘determination of liver left/right asymmetry’ and ‘thyroid gland development’ form a cluster of semantically similar terms with no genes overlap. This cluster shares overlapping genes with ‘determination of heart left/right asymmetry’ (highlighted in yellow)

Running the cardiac gene set with “Resnik similarity (average)” as similarity measure revealed that some GO terms cluster together despite having no overlapping genes (Fig. 7). Namely, the term ‘liver development’, ‘thyroid gland development’ and ‘determination of liver left/right asymmetry’ form a cluster even though there is no grey edge linking the neighbours. Thus, clustering by semantic similarity allowed us to identify two closely functionally related biological processes that are recruited in the formation of the heart, despite the lack of overlap in the genes sets composing these two processes.

**Fig. 8:**
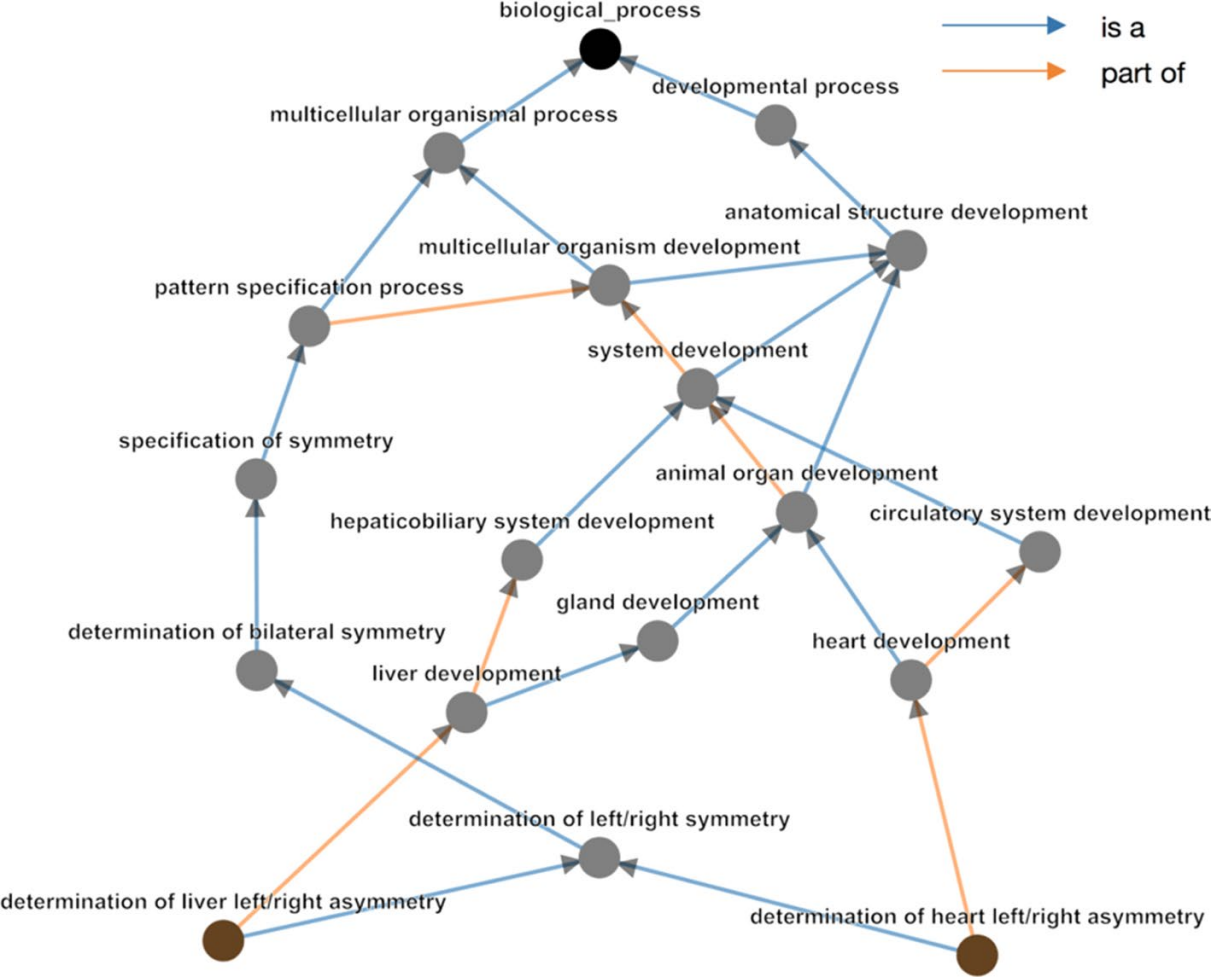
GO hierarchy between the Biological Process terms ‘determination of heart left/right asymmetry’ and ‘determination of liver left/right asymmetry.’ These have Resnik similarity of 4.229, with their Most Informative Ancestor being ‘determination of left/right symmetry.’

Since this gene set is found to be active in the heart of zebrafish, we further interrogated the functional link between liver and thyroid gland development (Fig. 7) and heart development. Neighbouring clusters in the chord diagram highlighted terms related to ‘left/right asymmetry’, including ‘determination of heart left/right asymmetry’. This suggests that heart, liver and thyroid gland development share common pathways during the determination of the left–right asymmetry of these organs. This common ancestor link is confirmed by the GO hierarchy (Fig. 8) and supported by biological evidence as ‘liver left/right asymmetry’ and ‘determination of heart left/right asymmetry’ show 20% of overlapping genes. Most importantly, the remaining genes that belong to the liver clustered and that do not overlap with the heart cluster are of great interest for the biologists. Indeed, clustering by semantic similarity allowed them to explore a novel hypothesis that genes belonging the liver term are novel genes involved in the regulation of heart left–right asymmetry. MonaGO’s implementation of two semantic distance measurements (Resnik, SimRel) provides a framework to cluster terms with optimal biological relevance and simplify the original input, even in the absence of previously known functional relationships.

### Expert evaluation

A case study was conducted to evaluate the user-friendliness, effectiveness and interpretability of the results presented by of MonaGO alongside the popular GO enrichment analysis tools DAVID [2] and Metascape [23]. Eight participants were asked to test the three tools by (1) analysing a curated zebrafish embryonic cardiac gene list (Additional file 1), (2) answering a questionnaire pertaining to the results obtained (Additional file 2), and (3) scoring each tool according to the criteria listed in Table 2 and Additional file 3. Half of the participants were researchers experienced in conduction of GO enrichment analysis while the others were using all three tools for the first time.

**Table 2:** Expert user’s perspective on the effectiveness of user interactions from three GO enrichment tools: MonaGO, DAVID, and Metascape

	MonaGo scoring	Metascape scoring	DAVID scoring
Required time to complete task	**4.5**	**3**	**3**
Relevance of outputs	**4**	**3**	**3.5**
Intuitiveness	**4**	**4**	**3**
Ease of use	**4**	**4**	**3**
Visual quality: layout and design	**4**	**3.5**	**2.5**
Sufficency of information	**4.5**	**2.5**	**3.5**
Customisability of resulting graphs	**4.5**	**3**	**2**
User friendliness	**4.5**	**3.5**	**3**

Eight expert users were asked to rate different aspects of the environments on a five-level scale: from most effective (5) to lowest quality (1). The median scores from eight participants were shown. The highest scores (including ties) based on each criteria (row) were highlighted in bold

MonaGO received the highest ratings of user-friendliness and intuitiveness (Table 2). 87.5% of the participants could answer all questions completely using MonaGO, which might be correlated with MonaGO achieving the highest median score (4.5/5) in the “Sufficiency of Information” category, providing help to achieve the tasks (Table 2).

While the median scores of ‘Ease of Use’ and ‘Intuitiveness’ were equal between MonaGO and Metascape, MonaGO median score was 1.5 points higher than both Metascape and DAVID when comparing the time required to complete the task. Furthermore, MonaGO’s features which allow the user to create custom graphs was positively received, emphasizing the improvements made by this tool compared to those already available.

In addition to the ratings listed in Table 2, participants were asked whether the output of each tool helped their understanding of zebrafish heart development. The evaluation of this question found that about 87.5% of participants using MonaGO answered this question with yes, whereas only about 12.5% for Metascape and 50% for DAVID answered with yes.

The case study revealed considerable overall satisfaction of the users using MonaGO as a GO enrichment data analysis tool. The user-friendly interface and intuitive use in connection with the provision of all information based on a meaningful representation of the data sets is especially valuable.

## Conclusions

MonaGO is a novel web-based visualisation with unique features enabling biologists with no programming knowledge to interactive explore the GO clustering hierarchy to rapidly deduce biological interpretations. To demonstrate the benefits of MonaGO using real-world problems from developmental biologists, our platform has shown novel biological insights that may have been overlooked using traditional non-interactive exploration of the GO hierarchy. Used in combination, MonaGO’s two distance measurements provide a framework to cluster terms with optimal biological relevance and simplify the original input, even in the absence of previously known functional relationships. As a result, MonaGO aims to provide a unique tool for biologists who are interested in hands-on interaction with the gene lists and their semantic relationship to derive biological interpretation.

## Availability of data materials

- Project name: MonaGO.
- Project home page: https://monago.erc.monash.edu; https://github.com/liyuanfang/MonaGO.
- Operating system(s): platform independent.
- Programming language: python, javascript.
- Other requirements: not applicable.
- License: GNU GPL.

## Supplementary Information

**Additional file 1. Test set. **Curated list of embryonic cardiac genes in zebrafish heart development.

**Additional file 2. Questionnaire for the expert user study. **Questionnaire used to compare MonaGO, Metascape and DAVID.

**Additional file 3. Complete results from the expert user study, Table S2.** The total numbers, aggregated for each score (1-5) for each criterion as collected from eight expert users were shown.

## Abbreviations

BiNGO: Biological networks gene ontology
DAVID: Database for annotation, visualization and integrated discovery
GO: Gene ontology
GOEAST: Gene ontology enrichment analysis software toolkit
GOplot: Gene ontology plot
GSEA: Gene set enrichment analysis
JSON: Javascript object notation
MonaGO: Monash gene ontology
PANTHER: Protein analysis through evolutionary relationships
PDF: Portable document format
PNG: Portable network graphics
REVIGO: Reduce and visualise gene ontology
SVG: Scalable vector graphics
UniProtKB: Universal protein knowledgebase
