# Supplementary material for: MonaGO: a novel gene ontology enrichment analysis visualisation system
Source: BMC Bioinformatics. 2022 Feb 14;23:69. doi: 10.1186/s12859-022-04594-1 (PMC8845231; doi:10.1186/s12859-022-04594-1)
Supplement: Supplementary file 2 — Additional file 2. Questionnaire for the expert user study. Questionnaire used to compare MonaGO, Metascape and DAVID. [file 12859_2022_4594_MOESM2_ESM.doc]

**Case study description**

**Project: ‘Comparative assessment of Gene Ontology enrichment analysis visualisation system’**

**Chief Investigator: A/Prof Mirana Ramialison**

You are invited to take part in this study. Please read this Explanatory Statement in full before deciding whether or not to participate in this research. If you would like further information regarding any aspect of this project, you are encouraged to contact the chief investigator, A/Prof Mirana Ramialison.

**What does the research involve?**

There will be 4 parts in the study.

1. Introduction

First, special terms used during the study will be explained, so that every participant has the same background knowledge.

2. Preparation

The selected software tools for gene ontology analysis in this study will be explained to the you.

3. Case study: zebrafish embryonic cardiac genes

One case study for investigating zebrafish heart development will be presented. A curated gene list known to be involved in zebrafish embryonic heart will be used. You will actively use the different software tools to achieve the biological goal.

4. Questionnaire

The questionnaire will be completed.

**1. INTRODUCTION**

**Gene ontology (GO)** provide a standardized vocabulary to describe genes and gene products from different species. GO terms allow us to assign functionality to genes. GO terms are hierarchical consisting of broader parent GO terms and narrower child GO terms. For example, “DNA replication” is a child of GO term: “cellular metabolic process”. “DNA replication” has child GO terms like “regulation of DNA replication”, “strand elongation”, *etc*. **GO enrichment analysis** is a way of summarizing the functions and biological categories of a specified list of genes.

**2. PREPARATION**

In this study, we will use three software tools for GO enrichment analysis: MonaGO, Metascape and DAVID.

1. MonaGO: <https://monago.erc.monash.edu/>

2. Metascape: <https://metascape.org/gp/index.html>

3. DAVID <https://david.ncifcrf.gov/tools.jsp>

**3. CASE STUDY: ZEBRAFISH EMBRYONIC CARDIAC GENES**

Zebrafish heart development is a biologically complex process, and we have provided an in-house curated list of zebrafish embryonic cardiac genes (see “Additional File 1.txt”). We used different GO enrichment analysis tools to assess which biological functions compose the developmental circuitry of the heart.

Input: a list of curated genes

Outputs:

- GO terms
- Similarity between these GO terms
- Biological annotations the GO terms

1. MonaGO: <https://monago.erc.monash.edu/>

- Identifier: “official gene symbol”
- Distance measurement: ‘percentage of overlapping genes’, ‘Resnik similarity (average)’


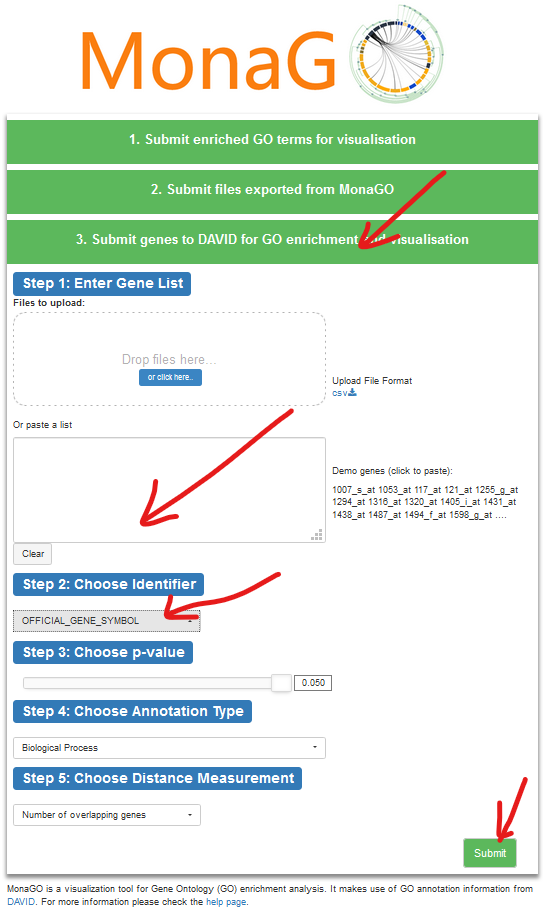


2. Metascape: <https://metascape.org/gp/index.html>


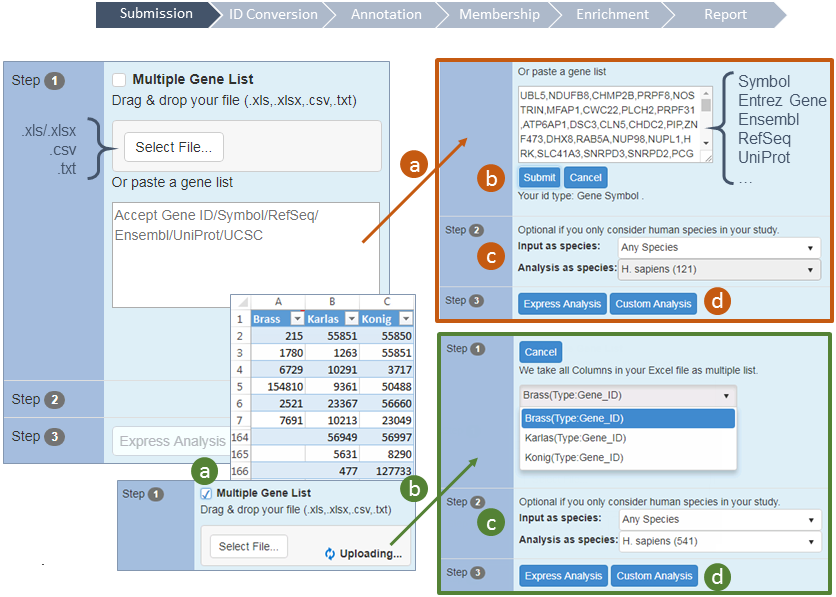


3. DAVID <https://david.ncifcrf.gov/tools.jsp>


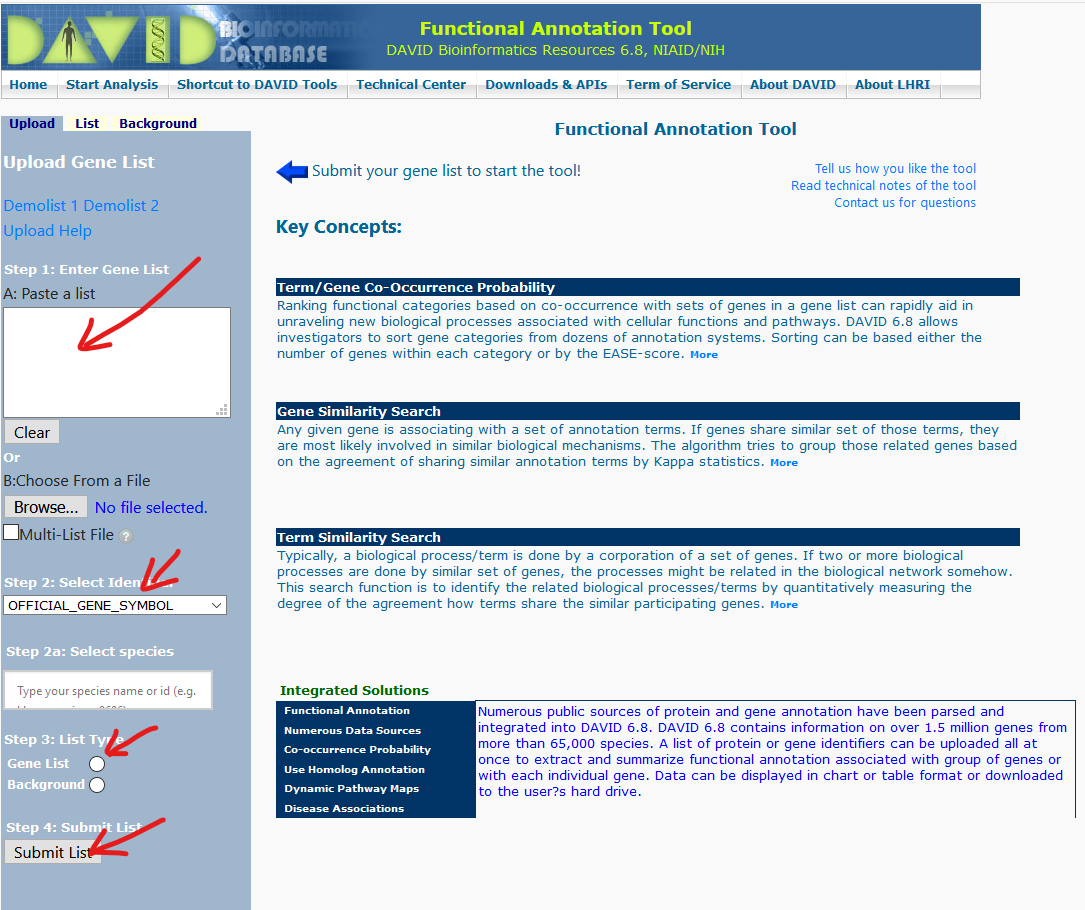


**4. QUESTIONNAIRES**

Please rate the provided GO enrichment analysis tools based on the case study.
**MonaGO**

Please tick (
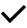
) the box:

1 – Very Poor

2 – Poor

3 – Neutral

4 – Good

5 – Very Good

| **Rating** | **1** | **2** | **3** | **4** | **5** |
| --- | --- | --- | --- | --- | --- |
| Time required to complete the task |  |  |  |  |  |
| Relevance of outputs |  |  |  |  |  |
| Intuitiveness / Easy to learn |  |  |  |  |  |
| Ease of use |  |  |  |  |  |
| Visual quality: Layout and Design |  |  |  |  |  |
| Sufficiency of information |  |  |  |  |  |
| Customisability of resulting graphs |  |  |  |  |  |
| User friendliness |  |  |  |  |  |

**Metascape**

Please tick (
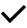
) the box:

1 – Very Poor

2 – Poor

3 – Neutral

4 – Good

5 – Very Good

| **Rating** | **1** | **2** | **3** | **4** | **5** |
| --- | --- | --- | --- | --- | --- |
| Time required to complete the task |  |  |  |  |  |
| Relevance of outputs |  |  |  |  |  |
| Intuitiveness / Easy to learn |  |  |  |  |  |
| Ease of use |  |  |  |  |  |
| Visual quality: Layout and Design |  |  |  |  |  |
| Sufficiency of information |  |  |  |  |  |
| Customisability of resulting graphs |  |  |  |  |  |
| User friendliness |  |  |  |  |  |

**DAVID**

Please tick (
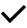
) the box:

1 – Very Poor

2 – Poor

3 – Neutral

4 – Good

5 – Very Good

| **Rating** | **1** | **2** | **3** | **4** | **5** |
| --- | --- | --- | --- | --- | --- |
| Time required to complete the task |  |  |  |  |  |
| Relevance of outputs |  |  |  |  |  |
| Intuitiveness / Easy to learn |  |  |  |  |  |
| Ease of use |  |  |  |  |  |
| Visual quality: Layout and Design |  |  |  |  |  |
| Sufficiency of information |  |  |  |  |  |
| Customisability of resulting graphs |  |  |  |  |  |
| User friendliness |  |  |  |  |  |
