# Supplementary material for: MonaGO: a novel gene ontology enrichment analysis visualisation system
Source: BMC Bioinformatics. 2022 Feb 14;23:69. doi: 10.1186/s12859-022-04594-1 (PMC8845231; doi:10.1186/s12859-022-04594-1)
Supplement: Supplementary file 3 — Additional file 3. Complete results from the expert user study, Table S2. The total numbers, aggregated for each score (1-5) for each criterion as collected from eight expert users were shown. [file 12859_2022_4594_MOESM3_ESM.doc]

| **MonaGo scoring** | **1** | **2** | **3** | **4** | **5** |
| --- | --- | --- | --- | --- | --- |
| Requried time to complete task | - | 1 | 2 | 1 | 4 |
| Relevance of | - | - | 3 | 2 | 3 |
| Intuitiveness | - | - | 1 | 4 | 3 |
| Ease of use | - | - | 2 | 3 | 3 |
| Visual quality: Layout and Design | - | - | 1 | 4 | 3 |
| Sufficency of information | - | - | 1 | 3 | 4 |
| Customisability of resulting graphs | - | 1 | - | 3 | 4 |
| User friendliness | - | - | 2 | 2 | 4 |
| **DAVID scoring** | **1** | **2** | **3** | **4** | **5** |
| Requried time to complete task | 1 | 2 | 2 | 2 | 1 |
| Relevance of | 1 | 1 | 2 | 3 | 1 |
| Intuitiveness | - | 3 | 4 | - | 1 |
| Ease of use | - | 2 | 2 | 3 | 1 |
| Visual quality: Layout and Design | 1 | 2 | 4 | 1 | - |
| Sufficency of information | 2 | 2 | 1 | 3 | - |
| Customisability of resulting graphs | 2 | 2 | 2 | 2 | - |
| User friendliness | - | 3 | 1 | 3 | 1 |
| **Metascape scoring** | **1** | **2** | **3** | **4** | **5** |
| Requried time to complete task | - | 3 | 4 | - | 1 |
| Relevance of | - | 1 | 4 | 1 | 2 |
| Intuitiveness | - | - | 2 | 6 | - |
| Ease of use | - | - | 3 | 4 | 1 |
| Visual quality: Layout and Design | - | 2 | 3 | 3 | - |
| Sufficency of information | - | 4 | 2 | 2 | - |
| Customisability of resulting graphs | - | 4 | 4 | - | - |
| User friendliness | - | - | 5 | 2 | 1 |
